# Virtual Staining-Enabled Colorectal Cancer Metastasis Detection in Liquid Cytology Based on Digital Holography

**DOI:** 10.3390/s25237272

**Published:** 2025-11-28

**Authors:** Yusen Gao, Xi Xiao, Ran Peng, Hao Wang, Feng Pan

**Affiliations:** 1 Key Laboratory of Precision Opto-Mechatronics Technology, School of Instrumentation and Optoelectronic Engineering, Beihang University, Beijing 100191, China; 2 Department of Radiation Oncology, Peking University Third Hospital, Beijing 100191, China; 3 Cancer Center, Peking University Third Hospital, Beijing 100191, China

**Keywords:** digital holography, colorectal cancer metastasis detection, liquid cytology, virtual stain

## Abstract

Colorectal cancer (CRC) remains a leading cause of cancer-related mortality, and detecting circulating tumor cells (CTCs) is crucial for early diagnosis and metastasis monitoring. Conventional staining-based cytology is costly, time-consuming, and often compromises sample integrity. In this study, we employed a combined digital holography (DH) and fluorescence imaging approach to develop a virtual staining framework for transforming quantitative phase imaging (QPI) data into interpretable pseudo-stained images. To the best of our knowledge, this is the first application of such a framework to colorectal cancer CTC detection. In our experiments, green fluorescent protein (GFP)-labeled HCT116 cells—generated through lentiviral transfection—were mixed with peripheral blood mononuclear cells (PBMCs) to create training datasets. The trained network achieved 99% classification accuracy and demonstrated strong generalization to unseen donors. This DH–fluorescence-based virtual staining method preserves cell integrity while enabling rapid, label-free, and low-cost liquid cytology diagnostics, highlighting its potential for non-invasive cancer detection and monitoring.

## 1. Introduction

Colorectal cancer (CRC) is the third most common malignancy worldwide and the second leading cause of cancer-related mortality, accounting for 10% of annual cancer diagnoses. Approximately 50% of patients experience metastasis during the disease progression [1]. Diagnosing metastasis in patients with metastatic colorectal cancer (mCRC) poses a significant clinical challenge. Conventional imaging techniques require the tumor to reach a certain size before detection [2,3]. However, many patients exhibit subtle clinical signs of metastasis, such as minor pleural effusion or ascites, complicating clinical diagnosis. Thus, analyzing circulating tumor cells (CTCs) in malignant pleural effusion, ascites, or even blood samples helps confirm colorectal cancer metastasis. Furthermore, CTCs are closely associated with poor prognosis, recurrence, and moderate predictive value, making their detection crucial for monitoring metastasis [4,5,6].

In the diagnosis of metastatic malignancies based on CTCs, common methods such as hematoxylin and eosin (H&E) staining [7] and immunocytochemistry [8] enable clinicians to distinguish cell types based on morphology and phenotype, but both have limitations. Conventional cytology diagnosis has low sensitivity, and inconsistent sampling procedures can lead to false negatives. Although immunocytochemistry is helpful, it lacks specificity, potentially producing false positives when benign cells express certain markers. Additionally, it requires specialized antibodies, making the process time-consuming and costly. Moreover, these traditional chemical staining methods demand skilled technicians for manual operation, and issues like photobleaching and phototoxicity affect imaging quality, rendering the labeled samples unsuitable for further biological testing.

The integration of quantitative phase imaging (QPI) with virtual staining technology now offers a novel approach for CTC detection. Quantitative phase images of unlabeled CTCs, obtained through QPI, can be transformed into easily interpretable stained images via virtual staining technology. This approach avoids the drawbacks of chemical staining methods while providing a reliable, label-free means of detecting CTCs.

In recent years, quantitative phase imaging (QPI) has found increasingly widespread applications in biological cell microscopy. QPI is a label-free imaging technique capable of capturing both the intensity and phase distribution of a target. The phase images obtained through QPI often exhibit higher contrast compared to intensity images, making this technique particularly effective for transparent objects like cells [9]. Among various QPI methods, digital holography (DH) has garnered growing attention. DH records an interference pattern (hologram) between a reference beam and the object beam, which can be numerically reconstructed to recover both amplitude and phase information of the specimen. This numerical reconstruction allows digital refocusing and three-dimensional visualization, making DH particularly powerful for analyzing transparent biological samples [10]. Compared to other QPI methods, such as differential phase microscopy (DPM) and spatial light interference microscopy (SLIM), DH provides full-field quantitative phase information, robust numerical processing capabilities, and flexible integration with existing microscopy setups [11]. Digital holography has been increasingly explored for biomedical cell imaging over the past two decades. In 2007, Moon and Javidi first demonstrated the three-dimensional identification of stem cells using computational holographic imaging, highlighting the potential of DH for volumetric analysis of biological samples [12]. Building on this foundation, Anand et al. (2012) employed digital holographic microscopy (DHM) with correlation algorithms to automatically identify malaria-infected red blood cells, showing the feasibility of DH for disease diagnostics [13]. Shortly thereafter, Watanabe et al. (2013) developed a high-precision microscopic phase imaging method that bypassed phase unwrapping, which significantly improved cancer cell identification using DH [14]. More recently, O’Connor et al. (2020) integrated deep learning with compact digital holographic microscopy to analyze spatio-temporal cellular dynamics, achieving accurate cell identification and disease diagnosis, and demonstrating the synergistic power of DH and artificial intelligence for clinical applications [15].

Despite the ability of QPI techniques, including DH, to generate quantitative contrast images of label-free objects, the current gold standard for cancer cell identification in clinical and research settings remains chemical methods, including staining. One major obstacle in applying QPI to cancer diagnosis is that most clinical practitioners are unfamiliar with these types of images and thus unable to interpret them effectively. Additionally, QPI lacks the cell specificity provided by external labels [16].

Thanks to advances in deep learning, virtual staining techniques now make it possible to directly identify cancer cells from quantitative phase images. Virtual staining refers to the use of trained deep neural networks to digitally generate stained images of tissues or cells, converting label-free images into versions that are more easily interpreted by medical professionals.

**Figure 1:**
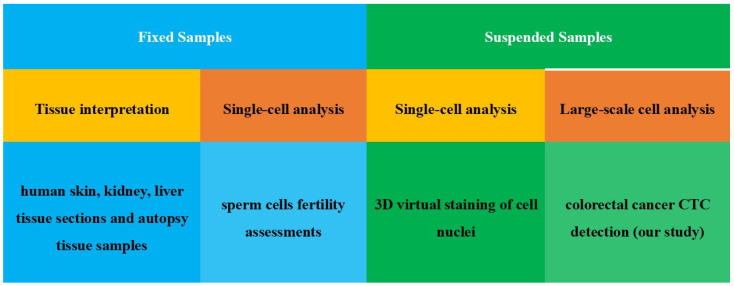
Research on Virtual Staining.

This method has been extensively explored by various research groups worldwide as shown in Figure 1. In 2019, Ozcan et al. trained a generative adversarial network (GAN) to perform virtual staining on QPI images of human skin, kidney, and liver tissue sections [17]. That same year, Nygate et al. developed virtual staining for sperm cells to assist in fertility assessments [18]. In 2022, Pietro et al. applied statistical principles to segment the nuclear region from the three-dimensional refractive index distribution of cells, enabling 3D virtual staining of cell nuclei [19]. In 2024, Ozcan et al. achieved virtual staining of severely autolyzed autopsy tissue samples [20]. The application of virtual staining technologies has facilitated the conversion of QPI images into stained images, aiding clinical diagnosis and medical research, and these techniques are being applied in an increasingly wide range of fields. We anticipate that virtual staining techniques will have similarly broad applications in the diagnosis of malignant tumor metastasis.

By integrating digital holography (DH) with virtual staining, we propose a liquid cytology-based method for detecting colorectal cancer metastasis. We use a dual-channel system combining digital holography (DH) and fluorescence imaging to acquire phase images and fluorescence labels from the same liquid cytology samples. The fluorescence images serve as training references, and the corresponding phase images are used as the inputs for virtual staining. To improve the staining accuracy, we also generate two orthogonal phase-gradient maps from the reconstructed phase distribution and include them as additional input channels. This combination of phase and gradient information enhances feature representation, facilitates the learning of cell boundaries and morphological differences, and enables reliable virtual staining of label-free DH images. While prior virtual staining studies—such as Nygate et al. [18]—focus primarily on isolated single-cell images, our work addresses a fundamentally different and clinically relevant problem: large-field liquid cytology analysis containing numerous PBMCs and rare CTCs. In this high-density environment, the combined use of phase and phase-gradient information helps differentiate cell boundaries and enhances rare-cell identification, which is not required in single-cell virtual staining tasks. Leveraging these advantages, our method achieves rapid, label-free, and accurate detection of circulating tumor cells (CTCs), offering a cost-effective and clinically viable solution for liquid cytology diagnostics.

For mixed samples of peripheral blood mononuclear cells (PBMCs) and GFP-labeled HCT116 cells, the trained U-net model can generate a virtual stained image with a resolution of 512 × 512 pixels, containing approximately 200 cells, in just 50 microseconds. The accuracy of cancer cell identification reaches 99.24%. Compared to traditional liquid-based cytology methods, such as hematoxylin and eosin (H&E) staining and immunocytochemistry, our method achieves high accuracy while offering lower costs, faster processing, and no requirement for chemical treatment of the samples. This feature facilitates further biological testing and presents a highly promising method for diagnosing malignant tumor metastasis.

## 2. Materials and Methods

### 2.1. Sample Preparation

HCT116 cells were obtained from Peking University Third Hospital and cultured in IMDM (Gibco, Grand Island, NY, USA) supplemented with 10% fetal bovine serum (FBS, Gibco), 50 IU/mL penicillin, and 50 µg/mL streptomycin (Gibco). The cells were maintained in a humidified incubator at 37 °C with 5% $CO_{2}$. GFP-labeled HCT116 cells were generated using standard lentiviral transfection procedures. Five blood samples were collected from healthy volunteer donors, who were researchers involved in this study, at the Department of Radiation Oncology, Peking University Third Hospital. Peripheral blood was drawn from veins into EDTA tubes (Sekisui Insepack^®^, Osaka, Japan) and processed within 6 h. Peripheral blood mononuclear cells (PBMCs) were typically isolated from whole blood using density gradient centrifugation. GFP-labeled HCT116 cells and PBMCs were separately counted and fixed with 4% paraformaldehyde for 15 min. The GFP-labeled HCT116 cells were mixed with PBMCs at ratios of 10:1, 50:1, and 1000:1. After thorough mixing, 0.1 mL of the simulated samples (containing 1–5 × 10^4^ cells) were spread on 10 × 10 cm^2^ glass slides for image scanning.

### 2.2. Imaging System

**Figure 2:**
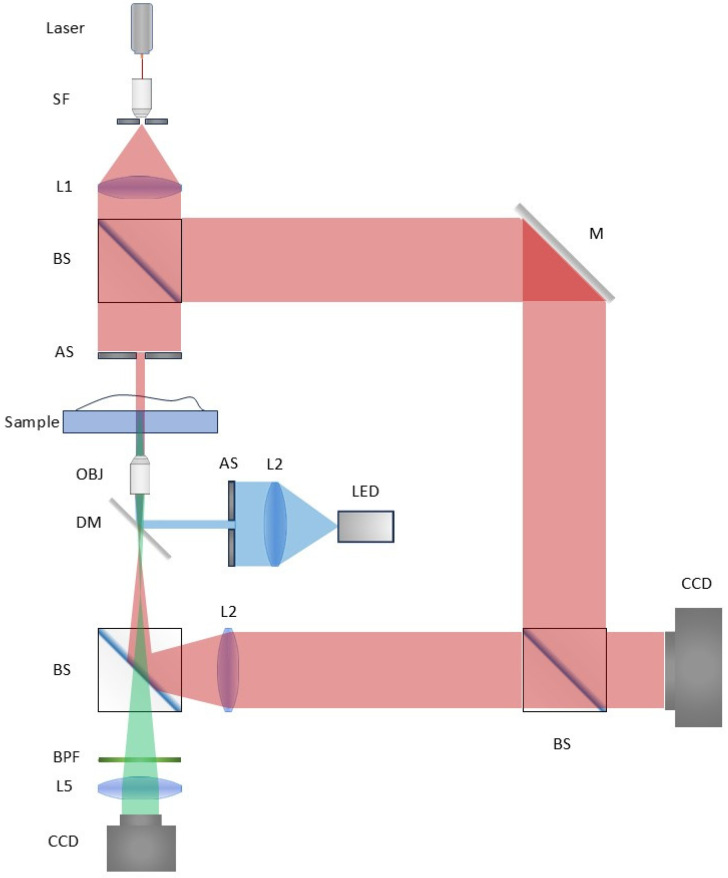
Optical setup of the experimental apparatus. (SF: Space filter, L: Lens, BS: Beam Splitter, AS: Aperture Stop, M: Mirror, BPF: Bandpass Filter, DM: Dichroic Mirrors).

In this study, quantitative phase images were acquired using an off-axis digital holography microscopy (DHM) system, whose schematic configuration is shown in Figure 2. The coherent illumination was provided by a 532 nm laser (Cobolt, Solna, Sweden) with an output power of 100 mW. The object beam was focused onto the sample through a condenser lens, transmitted through the specimen, and subsequently magnified by a microscope objective (Olympus UPLFLN 20×, NA = 0.50, Tokyo, Japan) combined with a tube lens to achieve optimal imaging resolution. The magnified object beam interfered with the reference beam at the sensor plane, and the off-axis holograms were recorded by a CCD (2048 × 2048 pixels, 5.5 μm pixel size, Point Grey, Canada). Fluorescence imaging was implemented in parallel with the DHM acquisition. The excitation light was generated by a broadband LED illumination module (GCI-060405, Daheng Optics, Beijing, China). The emitted excitation light was reflected by a dichroic mirror (Type-4 dichroic mirror, Zhaojiu Optics, Shanghai, China) toward the sample. The fluorescence emitted from the labeled cells passed through the same dichroic mirror and subsequently through a set of band-pass filters (BP466 and BP360, Zhaojiu Optics, Shanghai, China) to suppress excitation leakage. The filtered fluorescence was finally captured by a high-sensitivity scientific CMOS camera (SC2020, Zhongke Version, HeFei, China, 2048 × 2048 pixels, 10 μm pixel size), enabling high-fidelity ground-truth acquisition for training the virtual staining network.

### 2.3. Image Preprocessing

We employed digital holography (DH) to obtain the phase information of cells. The principle of DH phase imaging is as follows: when the object wave interferes with a reference wave on the camera sensor, the resulting hologram encodes both the amplitude and phase information of the object. To recover this information, the recorded holograms were reconstructed using the angular spectrum propagation method [21], which provides accurate numerical propagation and high-fidelity reconstruction. A numerical autofocusing procedure was implemented during reconstruction [22], in which a focus metric was evaluated across multiple propagation distances, and the distance yielding the maximum sharpness was selected as the optimal focal plane. To ensure that the reconstructed quantitative phase maps were free from system-induced distortions, phase aberration correction was performed by subtracting a background phase map [23]. A reference hologram was recorded in the absence of any sample under identical illumination and optical conditions.

After reconstructing the aberration-corrected quantitative phase distribution, we further processed the phase image to generate two differential phase maps. Specifically, the reconstructed phase map was shifted by one pixel in the x and y directions, respectively, and then subtracted from the original phase map. This pixel-shift subtraction provides robust approximations of the phase gradients in the orthogonal directions. Thus, through DH combined with this numerical processing, we obtain three outputs: (i) the original phase map and (ii) two orthogonal phase differential maps (x and y), which are subsequently used as input channels for the neural network.

On the other hand, the fluorescence images also require preprocessing. The raw fluorescence images captured directly have blurry cell edges and contain background noise, which would significantly impact the training performance if used directly as ground truth for network training. One of the main sources of noise in the fluorescence images is the residual fluorescence excitation light that the filter cannot completely eliminate, which illuminates the white blood cells in the image. Additionally, the blurry edges of cancer cells in the fluorescence images substantially reduce the network’s recognition accuracy. Furthermore, the blurred edges of the cancer cells make it difficult for the network input to properly align with them, complicating the network’s ability to accurately segment cancer cells. Therefore, preprocessing of the fluorescence images is necessary to address background noise and edge blurring. We generated masks from the fluorescence images to locate the corresponding cancer cells on the phase map, producing segmentation maps of the cancer cells in the phase images. Compared to the raw fluorescence images, these segmentation maps have clearer edges, cleaner backgrounds, and better alignment with the phase map and the two phase gradient maps. The ground truth for network training was the combined image of the segmentation map and the phase map.

### 2.4. Network

**Figure 3:**
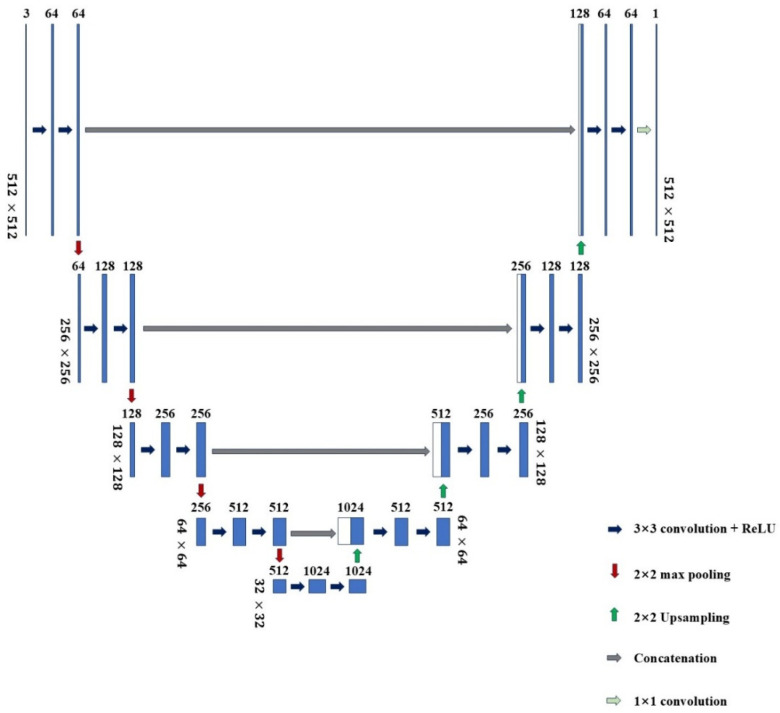
Network architecture.

We employed the U-net architecture as the model for network training. U-net is an end-to-end image segmentation model based on a convolutional neural network (CNN), characterized by its symmetric encoder–decoder structure, as shown in Figure 3. The encoder extracts image features, while the decoder reconstructs and generates the segmented image. This deep learning model is widely used for image segmentation tasks, particularly in biomedical image analysis. The encoder and decoder correspond to the downsampling and upsampling paths in the network, respectively. Each path consists of four blocks, forming four distinct hierarchical levels. The network input is a 512 × 512 3-channel image, and the output is a single-channel image of the same size. In the downsampling path, each residual block comprises two convolutional layers and two rectified linear unit (ReLU) activation functions, which are defined as:

(1) ReLU(x)=max(x,0)

At the output of each block, the number of channels is doubled. These blocks are connected by average pooling layers with a stride of 2, which downsample the output from the previous block by a factor of two in both the horizontal and vertical dimensions. In the upsampling path, each block also consists of two convolutional layers and two ReLU activations, but the number of output channels is reduced by a factor of four. These blocks are connected by bilinear upsampling layers, which upsample the output size from the previous block by a factor of two in both lateral dimensions. Skip connections are used to double the number of output channels by concatenating the corresponding feature maps from the downsampling path at the same level. The final layer is a convolutional layer that maps the output of the upsampling path to a single-channel image. The network’s loss function is Mean Absolute Error (MAE), which is defined as

(2) $$\mathrm{MAE}=\frac{1}{m}\sum_{i=1}^{m}\left|y_{i}-f(x_{i})\right|$$

Here, m represents the total number of data points, $y_{i}$ represents the ground truth for the ⅈ−th data point, and $f\left(x_{i}\right)$ is the predicted value for the corresponding input $x_{i}$. The MAE measures the average magnitude of errors between predicted and actual values, offering a more robust alternative compared to the Mean Squared Error (MSE) as it is less sensitive to outliers. The network is optimized using the Adam optimizer, a commonly used algorithm for deep learning that adapts the learning rate during training, ensuring faster convergence and reducing the likelihood of getting stuck in local minima. Training was performed on a desktop computer running Windows 11 (Microsoft, Redmond, WA, USA) with an Intel Core i7-13700KF CPU @ 3.40 GHz, 16.00 GB RAM, and an NVIDIA GeForce RTX 3070 GPU.

## 3. Results

**Figure 4:**
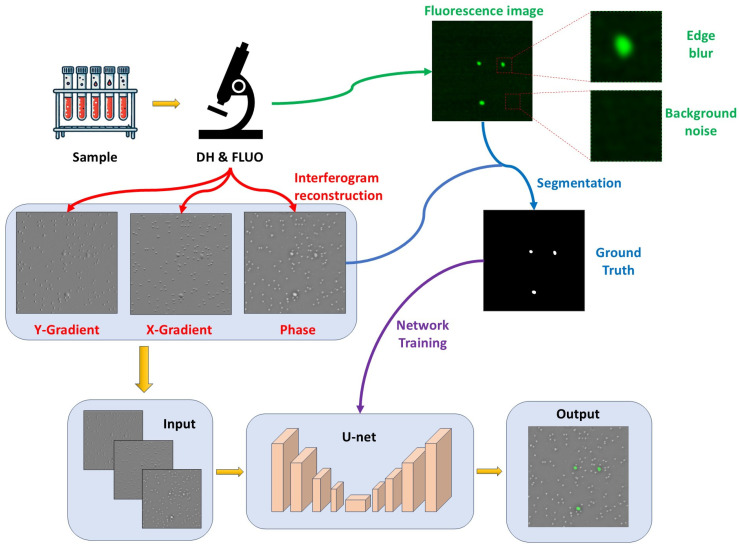
Workflow of virtual staining.

The workflow of this study is illustrated in Figure 4. Peripheral blood mononuclear cells (PBMCs) were collected from five healthy blood donors. PBMCs from each donor were mixed with GFP-labeled HCT-116 cells at three different ratios: 10:1, 50:1, and 1000:1. The primary purpose of the 10:1 and 50:1 ratios was to increase the proportion of cancer cells in the training samples, thereby improving the training performance. The 1000:1 ratio aimed to simulate the actual condition in circulating tumor cells (CTCs), where PBMCs vastly outnumber cancer cells. A total of 15 sample smears were scanned using a fluorescence microscope equipped with a digital holographic (DH) and fluorescence (FLUO) imaging setup. The acquired phase and fluorescence images were independently stitched into full-field maps by estimating inter-tile translations from matched cell-centroid positions in overlapping regions [24]; the resulting phase and fluorescence maps were then registered to each other using the same centroid correspondences to obtain pixel-level alignment. We then cropped image pairs with a size of 512 × 512 pixels at corresponding locations in both images, which served as the input and output for the network. For each donor and for each sample ratio, 100 images were cropped, resulting in a total of 1500 images.

**Figure 5:**
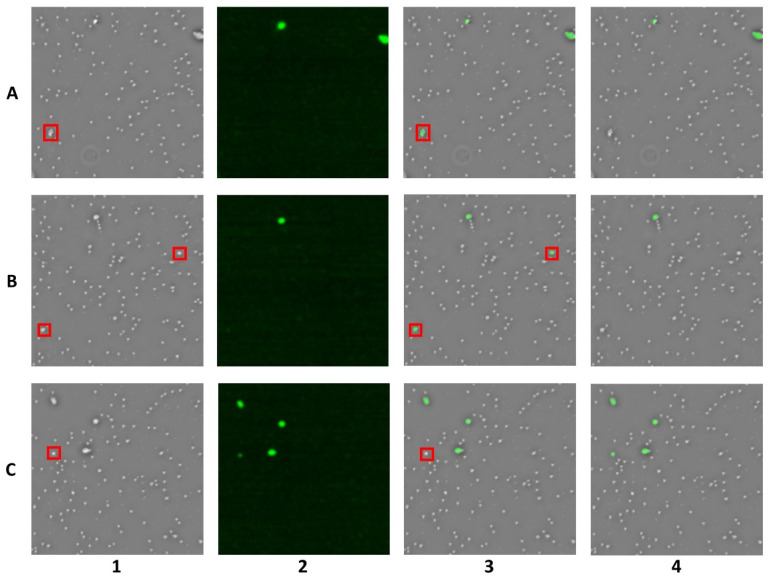
Comparison of staining results: phase-based vs. phase- & gradient-based. (1) phase map. (2) fluorescence image. (3) phase-based staining results. (4) phase- & gradient-based staining results. (**A**–**C**) samples exhibiting misclassification.

Based on the principles of image preprocessing, the reconstructed holographic images provide a phase map along with phase gradient maps in the horizontal and vertical directions. The gradient maps, compared to the phase map, emphasize the edges of cells more effectively. Combining the gradient maps with the phase map as network input helps the network better segment the cells, thereby reducing misclassification. Figure 5 illustrates the difference in network performance when using only the phase map as input versus using the combination of the phase map and gradient maps as input. When only the phase map was used as input, the network’s ability to segment closely adherent cells decreased. As shown in Figure 5A, the network misclassified four adherent white blood cells as cancer cells. However, with the addition of the gradient maps, the network correctly identified the adherent cells. Additionally, as shown in Figure 5B,C, the gradient maps improved the network’s accuracy in recognizing cancer cells, particularly those that are similar in size to white blood cells. The gradient maps helped the network more accurately distinguish cancer cells from white blood cells. Therefore, we combined the phase map and the two orthogonal gradient maps obtained through DH as input to the network to improve the accuracy of cell identification. The network input was a 512 × 512 3-channel image, as shown in Figure 4.

**Figure 6:**
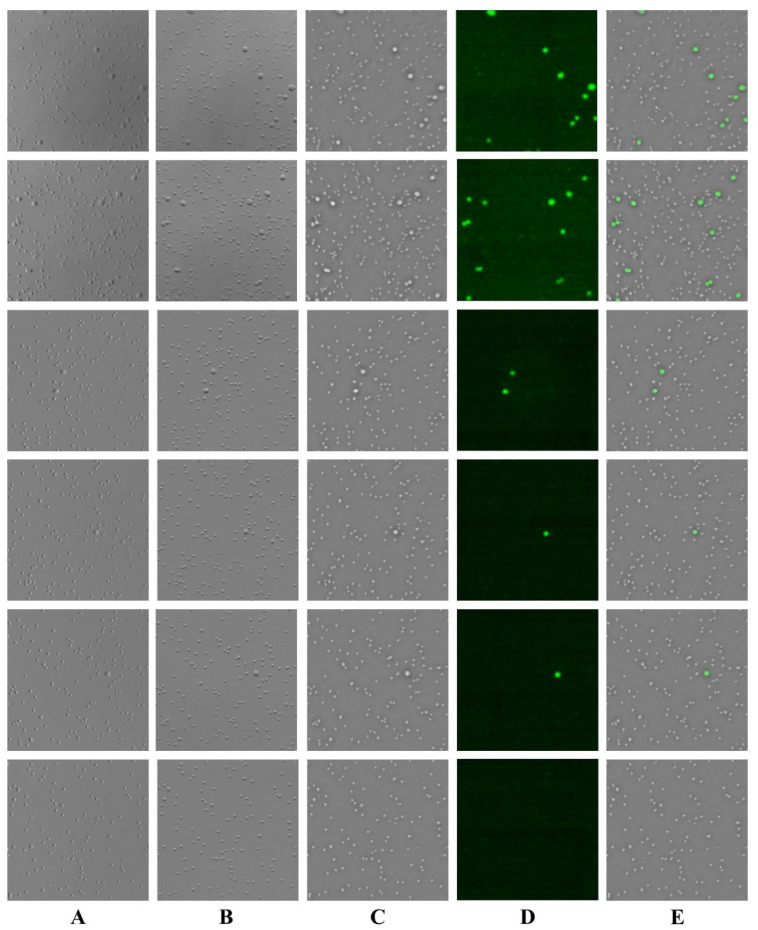
Virtual staining results for previously seen individuals. (**A**) horizontal phase gradient map. (**B**) vertical phase gradient map. (**C**) phase map. (**D**) fluorescence image. (**E**) network output.

In summary, we obtained a total of 1500 data batches, each consisting of a phase map, two gradient maps, and a cancer cell segmentation map. These batches were divided into training, validation, and test sets in a 3:1:1 ratio, and the U-net model was used for training. The input to the network was a 512 × 512 × 3 image composed of the phase map and the two gradient maps, while the ground truth for training was the cancer cell segmentation map. The network was trained using the 1200 image sets from the training and validation sets, and the virtual staining capability of the U-net was then evaluated using a test set of 300 previously unseen batches. Figure 6 presents some of the test results. Figure 6A shows the horizontal phase gradient map, Figure 6B shows the vertical phase gradient map, and Figure 6C shows the phase map—together, A–C form the network input. Figure 6D shows the fluorescence image, and Figure 6E shows the network output. The virtual staining results for samples with different cancer cell concentrations are presented in Figure 5. The first and second rows represent samples with a PBMCs:HCT116 ratio of 10:1, the third and fourth rows represent a 50:1 ratio, and the fifth and sixth rows represent a 1000:1 ratio. It can be seen that the network was able to accurately identify cancer cells across various concentrations.

For each of the 300 test images, we calculated the Structural Similarity Index (SSIM) between the output image and the ground truth, then averaged these values to obtain an overall SSIM of 0.9984. We believe that the training strategy incorporating gradient maps significantly improved the network’s output performance. For comparison, we set up one control group, where only the phase map was used as the network input, and the resulting SSIM was 0.9540. The strategy of adding gradient maps to the network input allowed the network to more clearly identify cell regions, thus improving the training performance.

**Figure 7:**
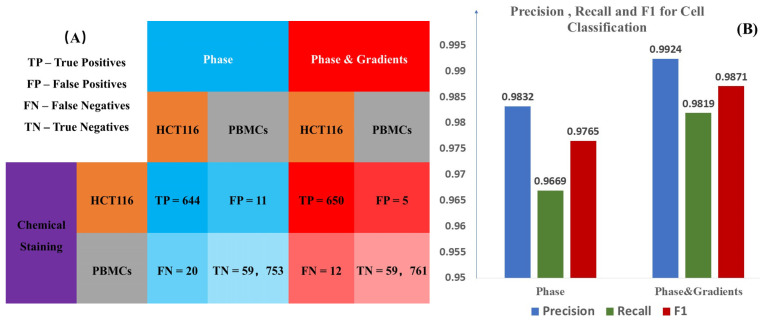
Statistical comparison of staining results using phase-based versus phase-and-gradient-based virtual staining approaches. (**A**) Table summarizes the cell count statistics obtained by the two methods. (**B**) bar chart compares their classification performance in terms of precision, recall, and F1-score.

To further evaluate the network’s performance, we conducted cell counting on the HCT-116 and PBMCs in the test set. Correctly classified HCT-116 cells were counted as true positives (TPs), while incorrectly classified HCT-116 cells were counted as false negatives (FNs). Similarly, correctly classified PBMCs were counted as true negatives (TNs), and incorrectly classified PBMCs were counted as false positives (FPs). The statistical results are shown in Figure 7.

In circulating tumor cell (CTC) detection from liquid samples, the accurate identification of cancer cells is considered critical. Therefore, achieving high precision for positive markers is essential, where precision is defined by Equation (3) below. Additionally, due to the disproportionate ratio of blood cells to cancer cells, a high recall rate (also known as sensitivity) is required, which is defined by Equation (4). Overall, the F1 score, defined by Equation (5), is calculated to quantify the balance between precision and recall for each dataset. The mathematical definitions of these three metrics are as follows:

(3) $$precision=\frac{TP}{TP+FP}$$

(4) $$recall=\frac{TP}{FN+TP}$$

(5) $$F1=2\times \frac{precision\times recall}{precision+recall}$$

For the two groups with different training strategies, we calculated the precision, recall, and F1 score of the network output, as shown in Figure 7. It can be observed that the use of gradient maps significantly improved the classification accuracy of both HCT116 cells and PBMCs. This improvement is particularly crucial for the detection of cancer cells, which are present in low concentrations in liquid samples.

**Figure 8:**
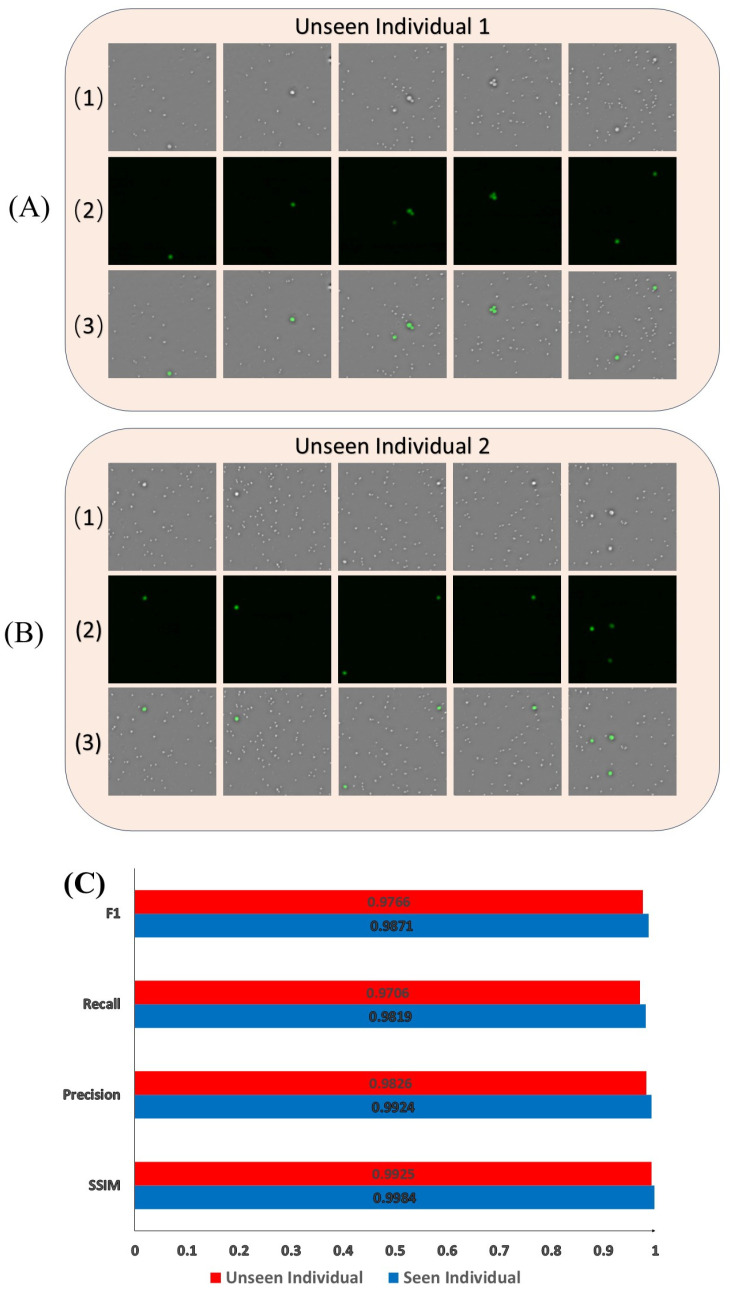
Virtual staining results and statistical comparison for unseen individuals. (**A**) unseen individual 1. (**B**) unseen individual 2. (1) phase map. (2) fluorescence image. (3) network output. (**C**) bar chart compares the classification performance in terms of SSIM, precision, recall, and F1-score between seen individuals and unseen individuals.

Additionally, to demonstrate the generalization ability of the model, we collected PBMCs from two additional healthy blood donors that the model had not previously encountered, and mixed them with HCT-116 cells to create samples. For these two unseen individuals, we collected a total of 600 images following the same ratios as before. After virtual staining by the network, we calculated the SSIM, precision, recall, and F1 score of the output results. The staining output and statistical results are shown in Figure 8. Both visually and at the cellular level, the results were comparable to those from the previously seen individuals. This indicates that the trained model can successfully perform virtual staining on samples from unseen patients, demonstrating that the model has strong generalization ability.

## 4. Discussion

Traditional staining methods remain the clinical gold standard but are time-consuming, costly, and may consume valuable samples. QPI, although label-free, produces images that cannot be directly interpreted by clinicians. By integrating digital holography (DH) with deep learning-based virtual staining, our method transforms QPI data into intuitive, stained-like representations, enabling rapid and low-cost cytological pre-screening.

Furthermore, our objective extends beyond virtual staining itself. We apply holographic virtual staining to colorectal cancer liquid cytology and demonstrate its ability to support downstream CTC identification across varying CTC–PBMC ratios (10:1 to 1000:1). To our knowledge, this represents the first application of DH-based virtual staining to colorectal cancer CTC analysis, addressing a clinically meaningful rare-cell detection scenario that differs substantially from previously reported tasks.

**Figure 9:**
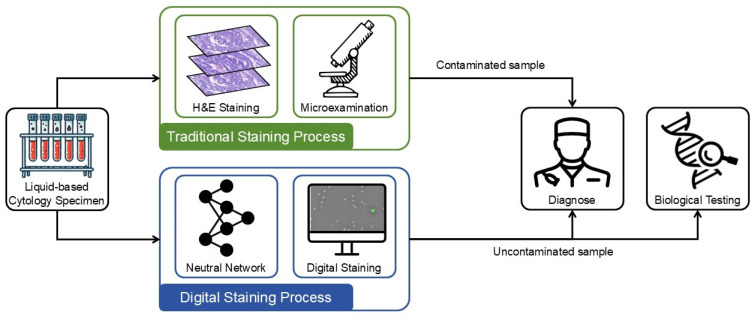
Comparison between virtual staining and traditional staining workflows.

As shown in Figure 9, this workflow enables non-destructive virtual staining on limited clinical samples, after which pathologists can review preliminary results and determine whether additional assays—such as immunohistochemistry or sequencing—are needed. This maximizes sample utility while improving diagnostic efficiency and reducing reagent use.

Overall, the proposed DH-based virtual staining framework advances prior work by addressing large-field multi-cell imaging, rare-cell identification, and clinical decision support, demonstrating strong potential for translation into liquid cytology diagnostics.

## Data Availability

The raw data supporting the conclusions of this article will be made available by the authors on request.
